# Functional Food—Consumer Motivations and Expectations

**DOI:** 10.3390/ijerph18105327

**Published:** 2021-05-17

**Authors:** Kinga Topolska, Adam Florkiewicz, Agnieszka Filipiak-Florkiewicz

**Affiliations:** 1 Department of Plant Product Technology and Nutrition Hygiene, Faculty of Food Technology, University of Agriculture in Krakow, 30-149 Kraków, Poland; agnieszka.filipiak-florkiewicz@urk.edu.pl; 2 Department of Food Analysis and Quality Assessment, Faculty of Food Technology, University of Agriculture in Krakow, 30-149 Kraków, Poland; adam.florkiewicz@urk.edu.pl

**Keywords:** functional food, consumer expectations, consumer motivations, consumer attitudes, consumer awareness

## Abstract

This review provides insight into consumer attitudes toward functional food (FF), with the purpose of better understanding the needs and behavior regarding this kind of product. A total of 47 articles were selected for this paper. The available studies from last 20 years differ according to the focus (awareness, attitudes, motivations, willingness, acceptance by consumers) and methodologies used. Several factors, including socio-demographic, cognitive and attitudinal ones, seem to be serve as the basis for the acceptance of functional products. The research papers showed that nutritional knowledge is the most important of these. Older people are more interested in functional products than younger consumers, because of their stronger belief in the health benefits of FF. Moreover, women are more open to compromise between taste and health properties. Claims concerning the disease preventative properties of FF are the most attractive for consumers. This review focuses also on future perspectives for the functional food market. Adequate knowledge and evidence-based communication seem to be the most promising ways to increase consumers’ interest in these kinds of products.

## 1. Introduction

Changes in lifestyle, including improper nutrition and inadequate physical activity, have resulted in the epidemic of non-infectious diseases being a cause of several health problems and even death [1].

Functional food (FF) influences specific functions of the organism, may provide (beyond basic nutrition) additional health benefits or remedy from some diseases following the addition/concentration of a beneficial ingredient, or removal substitution of an ineffective or harmful ingredient [2]. FF is defined as food products possessing the appearance of traditional food and included in the daily diet. These products provide physiological benefit and/or can reduce the risk of noncommunicable diseases. According to EU documents, if it can be proven that a food product affects one or more targeted functions in the body in a positive way, this food product is regarded as a functional food [3]. FF contains added ingredients, which provide health-related benefits for people beyond the effect of typical food products (not pills, supplements, etc.) [4]. At present, the functional product industry is an innovative one, characterized by dynamic growth, and new products are launched on a continual basis [1,5,6,7]. There are many positive health-related actions offered by this kind of food, including the potential to boost the immune system, to reduce the risk of (inter alia) cardiovascular problems, osteoporosis, obesity and cancer (some types) as well as to improve memory and physical condition [8,9,10,11,12,13,14,15].

Customers consider the various associated aspects, including potential benefits and risks, before deciding to buy a food product [16]. The success of FF depends both on its efficacy and ability to meet the demands of consumers [17]. This is why our review provides a report of research papers, with the purpose of better understanding the needs and behavior of consumers, regarding this kind of product. The changes in attitudes of consumers and—as a result—purchase decisions cannot occur without knowledge of their motivations and expectations. The scientific papers from the last 20 years on awareness as well as attitude of consumers towards this kind of food were examined using various databases. This review provides also some insights into future directions of the development of the functional food sector.

The list of publications on the subject of functional food is very long. We searched three databases: ScienceDirect, Pubmed and Google Scholar. The latter one, for example, has over 4 million records; the majority of them are review articles. This database contains not only scientific publications, but also dissertations, reports, abstracts, statements of various organizations/authorities, popular science papers, etc. The searching and evaluation of the three databases mentioned above showed that most of papers were concerned both with products and functional ingredients, in terms of technology, nutrition and health. A much smaller number of papers were related directly to consumers. Therefore, we aimed to focus on publications describing the research of FF in reference to the consumer. After the first review, we included articles having “functional food” and “consumer” in the title. There were 26 papers in ScienceDirect, six articles in PubMed and 94 items (80 papers published in 2000–2022) in the Google Scholar database (Table 1).

Exclusion criteria: documents, for which the full text could not be obtained; studies that are not published in English; opinions or statements; academic conference presentation materials and abstracts without full texts, theses, dissertations, editorials, duplicate articles.

The last search was developed on 6 February 2021. Ultimately, 47 articles were selected for this review.

### 1.1. Consumer Motivations, Attitudes and Willingness to Purchase Functional Products

In this chapter, we describe the main motivations and attitudes related to consumer choice of functional food. Because of the messages concerning functional product claims, this kind of food may lead to different consumer impressions [18]. The acceptance of a new product by consumers is important for market success, but the nature of this process is really multi-factorial [17]; better understanding could positively influence the marketing strategy.

One of the most frequently mentioned motivations is health [18]. Nowadays, in the third decade of the 21st century, health is becoming a more and more treasured value, both from a societal and a personal point of view. Regarding high costs of curative medicine, disease prevention is crucial [19]. There is evidence that FF consumers understand the role of this kind of product in maintaining good health (see Table 2).

Added to this, in the study of Urala and Lähteenmäki [20], among predictors of willingness to use FF, the best is perceived reward. The results of Goetzke et al. [19] also showed that health is a very important aspect for consumers of FF; however, their understanding of health was specific. The authors observed that functional food consumption was seen only as “small adjustments” to lifestyle. According to Çakiroğlu and Uçar [3], the factors most influencing the purchasing decisions of consumers were that “functional foods are necessary” and “functional foods are a part of healthy diet”. The longing for health as well as longer life are very effective factors; this was confirmed in several papers [5,6,18,19,21,22,23,24,25,26,27,28]. For example, Plasek et al. [1] attempted to identify the diseases that respondents wanted to avoid through the use of functional products in their diet. Most of the surveyed consumers were aware of the health risks associated with non-communicable diseases. Additionally, only a small group of respondents did not want to sacrifice money to prevent these health problems. A different viewpoint was presented in the research paper of Barauskaite et al. [29]. First, the authors stated that people concerned about their image in others’ eyes purchase FF for “good impressions” of their healthy lifestyle. Moreover, some of them may think that it is an effortless way, without the need for self-control or motivation, to replace appropriate diet, habits and exercise. Thus, these consumers—by choosing functional foods—can have the conviction that they are taking care of their health in a quick and easy way [29].

Besides the effect on health condition, sensory attributes (such as taste, flavor and texture) as well as convenience of use remain very important for consumers [30]. As a result of the survey of Kolbina et al. [31], among the residents of the city of Kemerovo, consumers pointed out both the FF potential in prevention or even treatment of some diseases and taste as the main criteria for theirs purchase decision. In the study of Urala and Lähteenmäki [20], there was a strong correlation between dimensions related to consumer attitude toward taste and to the rewards from FF consumption. Due to the strong health-related messages of some functional products, it was not a problem for motivated consumers to compromise on food taste. On the contrary, Çakiroğlu and Uçar [3] reported that in some studies consumers give “taste” particular importance in functional foods [32]. In the study by Williams et al. [33], taste and smell were perceived as attributes providing additional benefits for consumers. Complex consumer attitudes were also described by Gutkowska and Czarnecki [7]. Respondents, asked about the most important attributes of food, usually answered “that it has a healthy effect”; however, at the same time they prized the taste over the features related to health. The results obtained by Gautam et al. [2] indicated that beliefs about the link between nutrition and health, consumption patterns, and positive attitude towards FF significantly influenced the willingness to purchase.

Among the features of food products that are particularly important for consumers, packaging deserves special attention. In the study of Gutkowska and Czarnecki [7], consumers paid attention to the packaging (aesthetics as well as information placement). Therefore, packaging is an important factor in consumer perception and, consequently, FF purchase. Labels, providing information on the potential health benefits of functional products, could influence the purchase decisions of consumers to a high extent [6].

According to Williams et al. [26], attractiveness, uniqueness, and also credibility of food claims were responsible for only 56% of the intention to try. Thus, the two latter features of functional products enhanced purchase intent, but the extent was much smaller.

Another important feature for functional products is to be “reliable”. In the study of Çakiroğlu and Uçar [3], reliability (of the taste of product) was among the most important factors affecting consumer decisions. According to the paper mentioned above, the other factors that were found in literature to have impact on consumer motivations and attitudes toward functional foods were pleasure and awareness. Social trust, processing method and cultural values may also affect consumer willingness to use FF [5,13,25].

On the basis of Saher et al. [18], it should also be mentioned that people who buy functional products are regarded as more innovative (in comparison with consumers of conventional foods). However, the acceptance of innovation is specific, with skepticism about “improving” food (enrichment with various ingredients) yet positive attitudes to the reduction of components being unfavorable for health [7]. Bekoglu et al. [13] also showed that people who are innovative are more likely to consume functional foods.

The last but not the least important issue for consumer purchase decisions is the safety of functional foods. Indeed, some consumers could be suspicious about their “unnaturalness” [18]. This issue involves how consumers perceive the possible risks associated with the consumption of functional foods. Consumers who are convinced of the safety of FF are more willing to consume them [34].

Several socio-demographic factors, as well as cognitive and attitudinal ones, seem to be a potential basis for the acceptance of FF by consumers in the 21st century [22]. The reasons for their purchase and consumption are likely to be multi-factorial [30].

To sum up, available studies from last 20 years (see Table 2) differ according to focus (awareness, attitudes, motivations, willingness, and acceptance of consumers) and the methodologies used.

### 1.2. The “Portrait” of a Functional Food Consumer

One of the main observations on the basis of the reviewed papers, except for the definition of the criteria for FF purchase, is—in our opinion—the portrait of the consumer target group. Several studies in this area have been compiled in this chapter.

Among the factors influencing food choices, the following are of note: lifestyle, age, sex, personality, income, educational level, ethnicity, traditions, beliefs, physiological factors, and sensory preferences as well as marketing and available information (i.e., labels) [31,36,40,41,42,43,44,45,46,47].

One of the most important factors is the nutritional knowledge of the consumers. Indeed, in the study in [24], only consumers with the highest knowledge level were interested in product enrichment with ingredients such as fibre or antioxidants. Thus, appropriate strategies related to education may be needed to enhance FF consumption [24]. The study of Çakiroğlu and Uçar [3] also proved that education was connected with consumer interest in FF. According to Kolbina et al. [31], FF consumers are those aged 18-40 with higher education, for whom proper nutrition and product characteristics are important. These observations were in line with those obtained from the study in [24], related to healthiness perception and desire to use functional foods.

Based on the study of Verbeke [22], ‘‘benefit believers” are these consumers who faced illness in the family. Moreover, criticism of FF information is less intensive with ageing.

The next important factor differentiating attitudes of consumers is gender. Several studies [28,40,41] show the differences connected with dietary choices as dependent on gender. The explanation could be a greater participation of women in the control of body weight and their higher interest in healthy eating [41]. Knowing that men are often hard to reach through programs related to nutrition, another option could be functional products [18]. Moreover, the results of Hailu et al. [35] suggest that men prefer a pill more often than women, so consumer attitudes towards functional foods and nutraceuticals can be gender-related. In the study of Urala and Lähteenmäki [20], there were two subscales, and men and women scored differently. Women tended to doubt the possibility of counteracting an improper diet with the use of FF. They did not regard this kind of product to be as much a part of a healthy diet as compared to men. One of the main findings of Çakiroğlu and Uçar [3] was that the interest in FF was found to be high in women, university graduates and individuals aged 18-25. Decisions related to this kind of product were different according to gender as well as educational level (*p* < 0.05).

According to several studies [3,20,28,40,42], the age of consumers was also an important factor. Younger consumers were not convinced that they could improve their unhealthy diet with the use of FF. They were not ready to compromise on the taste of this kind of product for the health-related benefits [20]. It should also be noted that in the study of Çakiroğlu and Uçar [3], the average score related to individual purchase decisions was dependent (decreasing trend) on age. Additionally, according to the study of Ivkov et al. [42], people older than 50 years and people with fair/poor self-rated health status had more positive attitudes toward the healthfulness of pasta with inulin (compared to younger respondents and those with good and excellent health status). Respondents with an income above average levels had the most positive attitudes toward the price of FF (pasta with inulin as a functional food) [42]. Moreover, the results of Plasek et al. [1] also showed that gender did not have a significant impact on the consumer choice of functional products. The results from an Internet survey performed by Siegrist et al. [5] in Germany and China indicate that—among various factors—cultural factors have a special role in FF acceptance. Thus, caution is needed in generalizing research findings [5]. The consumer behavior related to food choice is a complex process, and several factors are important [17].

When trying to provide insight into the future of the functional food market, it should be remembered that consumers consider various risks before the purchase of a product. The lower the perceived risk is, the higher the consumer trend of online shopping [16].

Clear and transparent collaboration with nutrition and health specialists as well as product-specific marketing messages (based on scientific results) are of importance [27]. The attention of marketers should also be paid to the endorser in advertising [39]. It is known that the perception of risk is lower for the consumer in FF advertising with negative message framing as well as in high source-credibility advertising. This is why combined aspects (mentioned above) could be more effective in targeting consumer groups with rational motives [39].

Key trends concerning functional food products are announced every year. It is obvious that specific items on this list vary from year to year. There is, however, a consistent message concerning the future of functional foods. This future is dependent on demonstrated strong evidence on the health-related FF benefits for consumers. According to the trends in the food industry, FFs have become popular worldwide as a part of the daily diet [29].

## 2. Conclusions

Consumers in the 21st century have to face increased risks related to environmental pollution, stress, societal challenges and health problems. Functional products have the potential to help improve physical and mental health, leading to a higher quality of life.

On the basis on the review of the literature, it was shown that health benefits as well as motivation for use are the strongest positive determinants of FF acceptance. Women and elderly people are more interested and ready to compromise on FF taste for health. Independent of socio-demographic factors, inadequate nutritional knowledge could limit FF acceptance. It should be emphasized that health problems of family members increase consumers’ interest in functional products. People who regard FF as necessary products are perceived as innovative.

Because of the complex nature of consumer motivations and expectations, proper strategy for functional food design, technological development and marketing is crucial. Simultaneously, effective educational programs should be implemented. Since the definition of “functional foods” is differently understood worldwide, and this term is often abused, appropriate, evidence-based communication is strongly needed.

## Figures and Tables

**Table 1:** The results of the database search.

Key Words (in Title)	Year(s)	ScienceDirect *	PubMed *	Google Scholar **
Functional food	total	640	496	4670
	2000–2022	599	477	4480
Functional food AND consumer	total	26	5	94
	2000–2022	26	5	80

* Automatic advanced search: only research papers. ** After advanced searching (years of publication, “functional food and consumer” in the title), research papers were manually chosen.

**Table 2:** Factors influencing consumer attitudes toward functional food.

Author(s)	Aim	Respondents	Selected Findings
Saher et al., [18]	to apply an indirect measure to explore what kind of impressions people form of users of functional foods	350 Finnish respondents, 1 from 8 versions of a shopping list (healthy or neutral background items, conventional or functional target items) and rating the buyer of the foods (66 bipolar attributes on 7-point scales)	✓ buyers of FF regarded as more innovative; ✓ the impressions of FF users varied from conventional product buyers
Urala and Lähteenmäki, [20]	to quantify consumers’ attitudes towards so-called functional foods and to find the underlying dimensions	1158 respondents from all over Finland, mean age of respondents 44 years (range: 15–74 years), questionnaire related to food	✓ perceived reward from using and confidence in FF—the most important for consumers’ attitudes; ✓ the potential risk does not affect ratings describing willingness to use FF
Van Kleef et al., [21]	to examine the extent to which consumers perceive specific health claims associated with particular food products (one study); to examine how consumer responses to health claims are affected by various communication formats	50 Dutch respondents with an average age of 35.1; all of them had the primary responsibility for shopping in their households	✓ consumer evaluations vary primarily in relation to personal relevance of health claims; ✓ framing could be important, but the effect is dependent on health-related benefits
Verbeke, [22]	to investigate the role of socio-demographic, cognitive and attitudinal variables on the acceptance of functional foods	215 consumers from Belgium, functional food acceptance defined as giving a score of minimum 3 on a 5-point scale, simultaneously for acceptance in comparison to conventional counterpart	✓ belief in the health advantages of FF—main positive determinant of consumer acceptance; ✓ belief, knowledge as well as the presence of an illness in the family—potential determinants
Verbeke, [23]	to investigate socio-demographic and attitudinal determinants of consumer willingness to compromise on FF taste for health	two socio-demographically comparable samples from Belgium in 2001 (255 participants) and 2004 (205 participants), using a similar research method	✓ women and elderly people more ready to compromise on FF taste for health (in 2001); ✓ FF health benefit belief as the strongest positive determinant; ✓ significant increase in perceived importance of food for human health (from 2001 to 2004)
Ares et al., [24]	to evaluate the influence of nutritional knowledge on perceived healthiness and willingness to try functional foods	104 consumers from Uruguay, aged 18 to 81 years, 16 concepts consisting of combinations of carrier products and nutritional modifications	✓ consumers without adequate nutritional knowledge were not interested in FF purchase and use; ✓ fibre or antioxidants as added ingredients increased the willingness to try FF (for consumers with the highest nutritional knowledge); ✓ inadequate nutritional knowledge might be a limiting factor in FF acceptance
Siegrist et al., [25]	to examine factors that influence willingness to buy functional foods	a mail survey (*n* = 249) in Switzerland	✓ consumers are more interested in FF with physiological than psychological health claims; ✓ it is more probable that consumers trusting in the food industry will buy functional product; ✓ older participants more interested in FF
Williams et al., [26]	to compare the differences between consumers in health claims related to products with functional ingredients	149 participants from Australia, above 18 years	✓ claims concerning the prevention of serious diseases more attractive for consumers; ✓ differences observed in the attitudes of consumers from Australia as compared to similar study performed in Netherlands
Hailu et al., [35]	to explore the importance of each attribute in the preferences of products by consumers;to explore consumers’ socio-economic and behavioral variables	267 usable questionnaires foranalysis; Canada	✓ little value was placed by consumers on “non-verified” claims made by manufacturers of the products
Naylor et al., [36]	to explore the impact of beliefs in health claims of FF on the attitudes of consumers	Study 1: 178 students; United States of America; Study 2: 207 students; United States of America;	✓ consumers characterized by lower health consciousness (as compare to those with high consciousness)—sensitive to conflicting information concerning FF health claim validity
Del Giudice and Pascucci, [37]	to analyze the factors influencing the acceptance of functional foods of three distinct groups of young consumers	3 groups of 50 subjects each: Italian consumers with a humanities background, with a scientific background, young employees	✓ knowledge—the most important factor in FF acceptance by consumers; ✓ the impact of advertising of functional products on consumer attitudes is still minimal
Harrar de Dienes et al., [38]	to present a stage-wise application of AHP (Analytical Hierarchy Process) and CBC (Choice Based Conjoint)	Consumer surveys were conducted in Caracas, Venezuela; 5 food categories and 6 functional benefits were combined for a total of 30 concepts	✓ AHP and CBC as valuable techniques (simple, fast and unequivocal way) for the development of new product
Urala et al., [27]	to evaluate the awareness of the FF term, as well as consumption, consumer attitudes, and trust in information sources before food purchase decisions	32,800 invites with 1027 completes (from 546 counties in the United States of America)	✓ higher level of energy, boost of immune system, improving digestion—among the most important health concerns
Goetzke et al., [19]	to clarify the differences between consumers of organic and functional foods;to evaluate the effect of social desirability on consumer behavior	Two stages: a pretest (*n* = 40) and second test (*n* = 685); German consumers)	✓ health as an important aspect for both groups of consumers; ✓ different understanding of health; ✓ FF as a small “adjustment”
Soliha et al., [39]	to evaluate the role of message framing and source credibility	Selected adult participants (*n* = 278) from Indonesia, voluntarily chosen	✓ respondents feel lower risk perception in FF advertising with a negative message framing; ✓ combine aspects (message framing as well as source credibility)—more effective for people with a rational motive;
Siegrist et al., [5]	to examine willingness to buy FF and the influencing factors	Survey in Germany: *n* = 502; survey in China: *n* = 443	✓ Chinese consumers much more willing to buy FF than German ones ✓ higher willingness to buy FF among consumers with higher health motivation and more trust in the food industry; ✓ food neophobia in Chinese sample—a negative impact on acceptance of FF; ✓ cultural factors—a significant role in FF acceptance
Bekoglu et al., [13]	to evaluate the effect of attitude towards the necessity of FF consumption; to analyze demographic variables and their impact on FF consumption	695 responses by drop-off survey; Istanbul, Turkey	✓ people regarding FF as necessary, those being influenced by the others, and innovative ones—more likely to consume this kind of product; ✓ no differences in functional food consumption by men and women
Oliveira et al., [6]	to study consumers’ attentionto functional food labels, and to evaluate differences betweenregular and functional products (using probiotic milk as a case study)	60 respondents aged 18–45 y; recruited among students and workers, Uruguay	✓ the attention of respondents concerning labels decreased when the density of information was enhanced; ✓ graphic design as an important strategy to create health-related connotations
Grochowska-Niedworok et al., [40]	to analyze and evaluate the consumption of FF as dependent on several factors	300 respondents from Upper Silesia, Poland	✓ knowledge of functional food was not satisfactory; ✓ respondents preferred pharmacotherapy over dietary prevention;
Kraus et al., [28]	to determine the role of several factors in consumer purchase decisions as well as the most important motives for FF purchase	200 respondents from Holland (Netherlands), aged 18–60 y	✓ respondents with university education as well as women and older people valued mostly naturalness, freshness, safety, and nutritional value of food product; ✓ functional components more important for women; ✓ women as well as older men are more responsible for their health condition;
Küster-Boludaa and Vidal-Capilla, [41]	to study consumer attitudes toward FF	333 participants from Spain	✓ a healthy lifestyle had no significant effect on consumer attitudes; ✓ certain factors positively influence healthy lifestyle
Barauskaite et al., [29]	to reveal the relationship between conspicuous consumption, perceived self-control motivation, susceptibility to descriptive normative influence and the consumption of functional foods	900 respondents, aged 15–74 y; Lithuania	✓ relevance of social as well as hedonic motives for FF marketing and health promotion
Çakiroğlu and Uçar, [3]	to determine the attitudes of consumers toward purchasing at markets products that are suggested as functional food by nutritionists and dietitians	1182 respondents from Turkey, aged between 18 and 65, consumers shopping at big markets	✓ people want to consume FF because of the positive effects on health; ✓ consumers more willing to use FF when disease occurs more often
Gautam et al., [2]	to study the functional food market in “EASTERN UP” and understand the reasons and patterns behind consumer decisions to buy foods that could enhance their health	200 respondents in total, 6 districts of Eastern UP (Faizabad, Ambedkarnagar, Sultanpur, Basti, Jaunpur), 6 products surveyed	✓ beliefs about the nutrition and health link, current purchasing and consumption patterns, and positive attitude towards functional foods significantly affected willingness to pay
Ivkov et al., [42]	to evaluate the impact of the addition of inulin HPX on nutritional and sensory properties of spelt pasta;to evaluate the sensory performance, in terms of quality, of pasta with 20% inulin by inexperienced consumers; to examine consumer attitudes toward spelt pasta with inulin as a functional food	First part: instrumental examination of pasta with inulin Second part: sensory quality evaluation performed by 144 consumers from Romania. Third part: consumer attitudes; a total of 502 useable questionnaires were analyzed	✓ the presence of significant differences between consumer attitudes toward pasta with inulin as a functional food with regard to gender, age and income level; ✓ by using inulin HPX, it is possible to enhance the nutritional and sensory quality of wholemeal spelt pasta in a way that is acceptable by consumers
Petrescu and Petrescu-Mag, [43]	to contribute to understanding Romanian consumer behavior associated with rabbit meat (as FF);	a sample of 216 persons from Cluj-Napoca and from its surrounding localities (Romania)	✓ the awareness of behavior related to rabbit meat (being FF) is important for changing consumer behavior patterns
Plasek et al., [1]	to answer the question as to which prevention methods consumers would use to avoid/treat specific diseases	a survey with personal interviews with 1027 participants at busy transport hubs of five big cities in Hungary	✓ target group for each of 3 diseases was characterized; ✓ completed education—a key role in FF choice
Seccia et al., [44]	to evaluate the preferences of consumers related to table grapes	nationwide survey conducted in Italy	✓ “most important” features: product origin, chronic disease prevention, limiting of agro-chemicals use; ✓ brand or biodegradable packaging among features being “less important”
Rasanjalee and Samarasinghe, [34]	to investigate the influence of antecedents (Customer knowledge, Necessity, Safety, Confidence, Rewards) on the attitudes toward FF	280 participants, aged 18–60 y; Colombo district, Sri Lanka	✓ rewards having strong positive impact on the attitudes towards FF; ✓ high importance of claims on food label
Ribeiro et al., [17]	to assess consumers’ acceptance of farmed fish fortified with beneficial compounds;to comprise an assessment of fish consumption preferences	778 respondents (answered all questions); Portugal	✓ antioxidants and omega-3 fatty acids—the most accepted for fortification; ✓ appropriate communication is needed; ✓ farmed fish—a good candidate for FF
Gutkowska and Czarnecki, [7]	to identify consumers’ attitudes towards innovative food products and the sociodemographic profile of innovators on the food market as well as perceived and accepted changes	qualitative research using Focus Group Interview; Poland	✓ consumers accept innovations, but in different ways; ✓ consumers similarly perceive innovative and FF
Kolbina et al., [31]	to identify the demand for functional confectionery and to compile a target group of consumers	352 people from Russia, aged 18 to 70 years old, of whom 45% were men and 55% were women	✓ Internet and TV as the main sources of information related to beneficial properties of products; ✓ taste and prophylactic (or therapeutic) properties—main criteria for functional product purchase;
Nystrand and Olsen, [45]	to investigate antecedents of consumers’ attitudes and intentions to eating functional foods in a representativesample of consumers	an online survey in January 2019, 810 adult participants from Norway, 18–74 years, of whom 49% were female	✓ strong association between social pressure concerning the consumption of FF and consumer intention; ✓ improvement of FF hedonic attributes—possible benefit for food industry;
Papp-Bata and Szakály, [46]	to adjust health motivational models for consumers	focus groups; health-conscious and not health-conscious consumers from Hungary	✓ different marketing activities and actions should be used in these 2 groups of consumers;
Szakos et al., 2020 [47]	to examine health problems being a main concern for the respondents and to evaluate the acceptance of FF.	consumer survey conducted in 2018 (*n* = 1002), personal interviews were used; Hungary	✓ older consumers wanted FF to be integrated into a balanced diet; ✓ diabetes, cardio-vascular as well as digestive problems, and high cholesterol level in blood are to be remembered during FF designing for older consumers;
Quan et al., [16]	to provide insight into helping functional food customers and business managers to minimize e-commerce risks	500 respondents (374 volunteered to answer); Ho Chi Minh City, Vietnam	✓ customers’ (buying online) ideas and purchase intent related to belief about health benefits as well as the significance of sensory quality
